# Exploring the Relevance of Senotherapeutics for the Current SARS-CoV-2 Emergency and Similar Future Global Health Threats

**DOI:** 10.3390/cells9040909

**Published:** 2020-04-08

**Authors:** Marco Malavolta, Robertina Giacconi, Dario Brunetti, Mauro Provinciali, Fabrizio Maggi

**Affiliations:** 1 Advanced Technology Center for Aging Research, IRCCS INRCA, 60121 Ancona, Italy; r.giacconi@inrca.it (R.G.); m.provinciali@inrca.it (M.P.); 2 Mitochondrial Medicine Laboratory, Department of Medical Biotechnology and Translational Medicine, University of Milan, 20133 Milan, Italy; dario.brunetti@unimi.it; 3 Department of Translational Research, University of Pisa, 56126 Pisa, Italy; fabrizio.maggi63@gmail.com

**Keywords:** cellular senescence, viral infection, inflammation, mitochondria, extracellular vesicles, senoptotics, senolytics, SASP inhibitors

## Abstract

The higher death rate caused by COVID-19 in older people, especially those with comorbidities, is a challenge for biomedical aging research. Here we explore the idea that an exacerbated inflammatory response, in particular that mediated by IL-6, may drive the deleterious consequences of the infection. Data shows that other RNA viruses, such as influenza virus, can display enhanced replication efficiency in senescent cells, suggesting that the accumulation of senescent cells with aging and age-related diseases may play a role in this phenomenon. However, at present, we are completely unaware of the response to SARS-CoV and SARS-COV-2 occurring in senescent cells. We deem that this is a priority area of research because it could lead to the development of several therapeutic strategies based on senotherapeutics or prevent unsuccessful attempts. Two of these senotherapeutics, azithromycin and ruxolitinib, are currently undergoing testing for their efficacy in treating COVID-19. The potential of these strategies is not only for ameliorating the consequences of the current emergence of SARS-CoV-2, but also for the future emergence of new viruses or mutated ones for which we are completely unprepared and for which no vaccines are available.

## 1. Introduction

The global outbreak of severe acute respiratory syndrome coronavirus (SARS-CoV) in 2003 and the more recent coronavirus 2 (SARS-CoV-2) have made it clear that older people, especially those with comorbidities, die more easily from these infections than younger people [1,2,3]. The global spread of the novel coronavirus disease (COVID-19), and the emerging evidence of a sustained human-to-human transmission, suggest that we are dealing with a historic challenge to our capacity to protect the health of our elderly community.

This pandemic requires concerted action in all sectors of public health, but it also poses a challenge for biomedical aging research. Indeed, understanding the biological mechanisms that are the basis of the higher susceptibility to death in elderly people may be important to plan preventive and therapeutic strategies in the current pandemic, as well as for the future emergence of similar pandemic viruses for which vaccines are unavailable.

## 2. Mechanism Driving Inflammation during SARS-CoV-2 Infection

A reduced proportion of lymphocytes and an increased inflammatory response have been observed in the elderly with the new-type of SARS-CoV-2 pneumonia [4]. The novel coronavirus has been reported to use the same receptor, angiotensin-converting enzyme 2 (ACE2), used by SARS-CoV, and spreads mainly through the respiratory tract [5,6]. However, this seems not to be the only target used by coronavirus to attack human host cells. Glucose regulated protein 78 (GRP78) [7] and furin [8] have also been identified as membrane proteins that mediate the viral invasion. Once inside cells, the viral RNAs are detected by the pattern recognition receptors (PRRs) which trigger a downstream cascade of molecules leading to the activation of the transcription factor nuclear factor-κB (NF-κB) and interferon regulatory factor 3 (IRF3), with the subsequent production of type I interferons (IFN-α/β) and a series of pro-inflammatory cytokines [2,9] including interleukin (IL)-1β and IL-6 [10]. The development of an uncontrolled inflammatory response can thus lead to potentially life-threatening damage to lung tissue.

For this reason, a clinical trial with a monoclonal antibody against the IL-6 receptor (tocilizumab, also known as atlizumab) has recently started in China in COVID-19 patients (ChiCTR2000029765). Tocilizumab is an immunosuppressive drug, mainly used for the treatment of rheumatoid arthritis (RA) and systemic juvenile idiopathic arthritis. Other drugs with similar targeting, such as TZLS-501 (Tiziana Life Science, London, UK) are also in course of development.

Hence, some of the proposed, putative therapies for COVID-19 to limit death in elderly (comorbid) patients are based on inflammation targets. However, increasing evidence points to the accumulation of senescent cells in different body tissues as the main source of inflammatory components during aging and age-related diseases [11]. Using network proximity analyses of drug targets and CoV–host interactions in the human interactome, additional anti-HCoV repurposable drugs have been identified [12]. Among them, rapamycin (sirolimus) is a specific inhibitor of mTOR that can promote autophagy and suppress the secretory phenotype of senescent cells [13]. Another proposed drug candidate is melatonin which was shown to display similar effects on the senescent secretory phenotype of lung fibroblasts [14]. A bioinformatic approach supported by artificial intelligence (AI) has identified ruxolitinib among the potential therapeutics [15]. This is a potent and selective JAK inhibitor approved for rheumatoid arthritis and myelofibrosis. It has been recently proposed as a potent suppressor of the secretory phenotype of senescent cells [16,17] and is currently being tested in at least one clinical trial for COVID-19 (NCT04331665). In contrast, chloroquine, an autophagy inhibitor, was shown to promote senescence in endothelial cells [18], and is currently undergoing testing in clinical trials for COVID-19 [19]. Chloroquine or its safer analogue hydroxychloroquine, is deemed to display enhanced effect when azithromycin is included in the treatment (NCT04322396). However, another pilot study found no evidence of antiviral clearance or clinical benefits with this combination [20]. The purpose of the combination of hydroxychloroquine with this antibiotic is mostly to prevent bacterial superinfection, but recent literature has identified azithromycin among the compounds (currently named senolytic drugs) which selectively kill senescent cells [21]. Importantly, it was reported that azithromycin presents more general antiviral effects, as shown for Zika virus [22] and influenza A(H1N1)pdm09 virus [23]; moreover it is recommended for the treatment of chronic airways diseases, such as cystic fibrosis [24] and chronic obstructive pulmonary disease [25], in which cellular senescence has been proposed as a key pathological mechanism [26,27]. Current therapies proposed for SARS-CoV-2 involving cellular senescence are reported in Table 1.

In light of this evidence from preliminary therapeutic findings, we deem that the interaction of senescent cells with coronaviruses deserves particular attention in the development of therapies for SARS-CoV, SARS-CoV-2 and future emerging viruses.

## 3. Cellular Senescence and Inflammatory Response

Cellular senescence entails a multitude of processes which culminate in a state of irreversible cell cycle arrest in which cells undergo distinctive phenotypic alterations, including profound chromatin and morphological changes, increased lysosomal activity and levels of tumor-suppressors, including p53, p16 (Cdkn2a), and p21 (Cdkn1a), as well as significant secretome changes [30]. Cellular senescence is considered to be an important anti-cancer mechanism as well as a physiological process for tissue regeneration. However, the excessive accumulation of senescent cells observed in aging and age-related diseases seems to contribute to chronic inflammation as well as to tissue and organ dysfunction.

Hence, it is reasonable to hypothesize the involvement of cellular senescence in the increased death rate of COVID-19 among elderly (comorbid) patients. Drugs targeting IL-6 have been included among the potential strategies to inhibit the deleterious consequences of the senescence-associated secretory phenotype (SASP), the secretome produced by senescent cells [31,32]. The SASP includes cytokines, chemokines, proteases, and growth factors, recently collected in a proteomic database [29], which functionally links the accumulation of senescent cells with its pathological processes [33,34]. However, the SASP appears to be beneficial or deleterious, depending on the biological context. For example, the SASP is also responsible for activating an immune surveillance response to remove senescent cells [35]. This response seems to involve macrophages, T-cells as well as NK-cells, and appears to differ in different tissues. In particular, an orthotopic transplantation model of senescence-inducible tumor cells in mice has provided evidence that, in contrast to the phenomenon observed in the liver [36], NK-cells may limit the efficient clearance of senescent cells in the lung [37]. Although this observation may suggest the hypothesis that senescent cells easily accumulate in aged lungs, understanding the extent and kind of senescent cell accumulation in aging lung tissue is still a challenge. A recent survey of senescent cell markers (presence of p16- and p21-positive cells) found an increased number of p21-positive and p16-positive cells with donor age in skin (epidermis), pancreas, and kidney, but not in lung [38]. However, senescent fibroblasts, smooth muscle cells, and/or alveolar epithelial cells have been implicated in the etiology or progression of several human diseases, including chronic obstructive pulmonary disease, idiopathic pulmonary fibrosis and emphysema [39]. In mice, aging does not contribute to the induction of cellular senescence by cigarette smoke in the lung of a mouse model of COPD/emphysema [40], but other studies using aged wild type mice display an increase of p16Ink4a mRNA and senescence-associated-β-galactosidase (SA-β-Gal) activity in the lung compared to young controls [41,42]. The stimuli that drive lung cells into senescence are still incompletely understood and may include infection or inflammation due to infection. Notably, senescent cells are not only a source of inflammatory mediators, but they are also carriers of additional damage through a contagious spreading of senescence and inflammation in neighboring and even distant tissues [43]. The inflammatory components of the SASP are not the only player in this phenomenon. Most recent evidence has demonstrated that the SASP includes extracellular vesicles (EVs) through which senescent cells exert central effector functions in the local environment. Senescent cells secrete EVs with a distinctive, but still incompletely characterized, content of miRNA, proteins, and DNA that can spread senescence in surrounding and even distant tissues [44,45,46,47], thus promoting further inflammation and catastrophic consequences for the organism [48]. Interestingly, it has been documented that exosomes are crucial components in the pathogenesis of virus infection, but specific studies for SARS-CoV or SARS-CoV are still lacking [49].

## 4. Cellular Senescence and Response to Viruses

Importantly, cellular senescence has been also described as an anti-viral mechanism [50]. Indeed, the cell cycle arrest and the release of proinflammatory cytokines and chemokines that characterize cellular senescence are strikingly similar to the features observed by many cells during an antiviral response. Senescence can also be induced by prolonged signaling of cytokines, such as β-interferon, in response to viral infection [51] and the SASP can induce senescence in neighboring cells [52] which have a high probability of being infected. Moreover, many viruses encode inhibitors of programmed cell death to subvert the host responses during infection [53], suggesting that chronic viral infection may contribute to the resistance to apoptosis hypothesized for some senescent cells that accumulate with aging [30]. This hypothesis was also confirmed by the observation that the replication of vesicular stomatitis virus is impaired both in primary and tumor senescent cells in comparison to non-senescent cells [54]. Many studies have described a higher prevalence or increased circulating viral load of some viruses, such as cytomegalovirus (CMV) [55] and Torquetenovirus (TTV) [56,57], with aging. The increased grade of inflammation associated with this phenomenon and the association with mortality of TTV and CMV in the elderly highlights the possible involvement of cellular senescence. In this context, TTV viremia can predict CMV reactivation [58] and a mechanistic link between CMV and accumulating senescent cells has already been proposed [59]. The CMV serostatus plays also a prominent role in determining the magnitude of response to influenza vaccination [60].

Some viruses have been shown to induce cellular senescence, such as human immunodeficiency virus-1 [61], Epstein–Barr virus [62], measles virus [63] and dengue virus [64]. Similar results have been obtained using the virulence factor, NS1 protein, of the influenza A virus (IFV-A) [65]. Other viruses have developed mechanisms to overcome senescence and to evade anti-viral response, as reported for simian virus 40 [66] and human papillomavirus [67]. More recently, it has been shown that IFV and Varicella Zoster Virus display enhanced replication efficiency in senescent human bronchial epithelial cells as well as in senescent human dermal fibroblasts compared to non-senescent cells [68].

Coronaviruses are similar to IFVs, in that they both are RNA viruses, but we are completely unaware of the types of interaction and type of response that SARS-CoV or SARS-CoV-2 can display in senescent cells. Regarding the molecular targets of SARS-CoV-2 virus, there is no evidence that these can be upregulated in senescent cells, and some studies may eventually suggest that ACE2 [69] as well as for GRP78 [70] are downregulated by senescence. However, in few months of studies of COVID-19 pathogenesis, at least four routes of entry have been identified and it is unlikely that the reduced expression of one or two of these targets may prevent infection of senescent cells. Conversely, it should be urgently addressed if the results observed for normal IFVs (i.e., that they can induce senescence and can use senescence to increase their replication rate) can be extended also to SARS-CoV and SARS-CoV-2. If this is proven to be true, it could be reasonable to hypothesize that the massive presence of senescent cells in elderly and comorbid patients may exacerbate the mortality rate. The evidence that patients with cancer, hypertension or with smoking habits (conditions associated with a pathological role of cellular senescence) experienced worse outcomes from COVID-19 [71], further supports the hypothesis that an accumulation of senescent cells may favor the development of severe events during SARS-CoV-2 infection.

Spreading of senescent cells through the organism is limited by immune surveillance [72,73,74]. Impaired immune surveillance of senescent cells may contribute to their accumulation during aging [75] and to the development of cancer [74]. Some component of the SASP contributes to attract and activate immune cells, thus suggesting that alteration of this process could be particularly important in the case of viral infection. Age-associated changes in CD4 T-cell functionality have been linked to chronic inflammation and decreased immunity [76] as well as to senescence immune surveillance [74,77]. Depletion of CD4 T cells resulted in an enhanced immune-mediated interstitial pneumonitis and delayed clearance of SARS-CoV from the lungs [78].

There are also mechanisms that some viruses have evolved to overcome immune surveillance of infected cells, for example by targeting mitochondria.

Mitochondria dysfunction in senescent cells can drive important and even specific alterations of the SASP [79]. When the mitochondrial quality control system is impaired, mitochondria could release into extracellular space various components (including formyl peptides, mitochondrial DNA, TFAM, cardiolipin, ATP, succinate and cytochrome C) that could promote or exacerbate SASP; this phenomenon is named mitochondrial damage-associated molecular patterns (DAMPs) [80]. Many models of senescent cells are associated with a shift of mitochondria toward more hyperfusion events, resulting in the presence of abnormally elongated mitochondria [81]. It was also demonstrated that the reduction in mitochondrial content by mTOR inhibition prevents senescence and attenuates the SASP [82].

**Figure 1:**
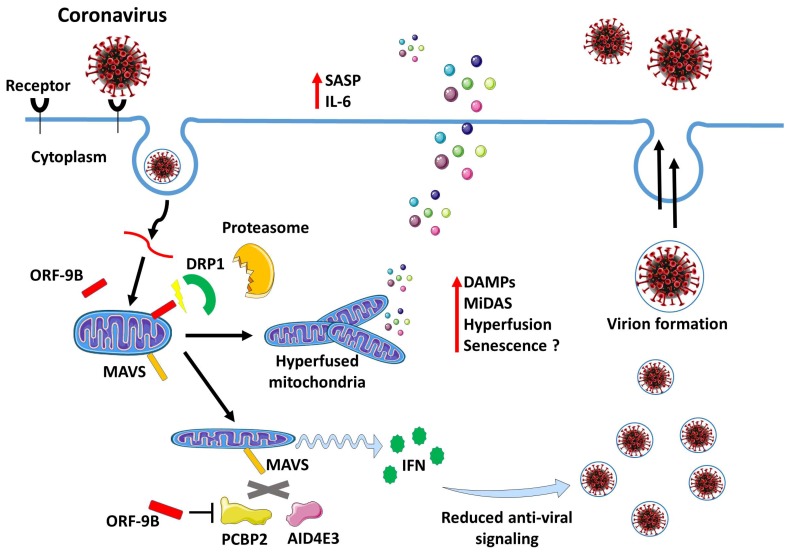
Modulation of mitochondrial dynamics upon CoV Infection. ORF-9b, a virulence factor of severe acute respiratory syndrome coronavirus (SARS-CoV), localizes to mitochondria and causes mitochondrial elongation by triggering ubiquitination and proteasomal degradation of dynamin-like protein (DRP1). Further, acting on mitochondria ORF-9b targets the mitochondrial-associated adaptor molecule MAVS signalosome by usurping poly(C)-binding protein 2 (PCBP2) and the HECT domain E3 ligase (AID4E3) to trigger the degradation of MAVS. This reduces host cell interferon responses and antiviral signaling. However, these changes may later trigger cellular senescence and contribute to enhance the inflammatory response via the senescence associated secretory phenotype (SASP).

A mechanism based on mitochondria alterations was proposed to explain how SARS-CoV can escape innate immune surveillance. A protein encoded by SARS-CoV designated as open reading frame-9b (ORF-9b) localizes to the mitochondria, where it promotes proteasomal degradation of Drp1, a protein involved in mitochondrial fission [83], leading to mitochondrial hyperfusion [84], which is a common phenotype of senescent cells and a mechanism involved in resistance to apoptosis [85,86]. The SARS-CoV ORF-9b provides a receptive intracellular environment for viral replication by targeting the mitochondrial antiviral signaling protein (MAVS) signalosome [84]. During the SARS-CoV infection, in the presence of ORF-9b, MAVS undergo degradation and this process is accompanied by the loss of TNF Receptor Associated Factor 3 and 6, two other key signaling intermediaries in antiviral defenses. This leads to an impairment of the host cell IFN responses [84]. However, silencing of MAVS induces a general repressive action on NF-κB with the subsequent suppression of IL-6 expression in senescent cells [87]. This may eventually favor viral replication in the initial phases of the infection without alerting the immune system. In agreement, a significantly enhanced production of IL-6 is not detected until day 4 after infection of human lung epithelial cells with SARS-CoV [88]. This could be consistent with an initial suppression of the mitochondrial antiviral response followed by the later development of a senescent-like phenotype (Figure 1). Hence, it should be important to verify if SARS-CoV and SARS-CoV-2 may induce alteration of the SASP in senescent cells through a similar process.

Mitochondrial fusion is generally required for intracellular proliferation of the viruses and evasion of the antiviral innate immune signaling, as demonstrated for the dengue virus infection mechanism [89]. In the case of dengue virus infection, cellular senescence seems to exert an anti-viral function [64] but other viruses, such as IFV, have evolved mechanisms to increase replication in senescent cells [68]. These observations illustrate that the behavior of different viruses in response to senescence can be different and that specific studies for each virus are needed.

## 5. Therapeutic Perspectives

Clarifying the role of cellular senescence in SARS-CoV infection may additionally provide a strong rationale for the use of senotherapeutics, such as SASP inhibitors, in the management of elderly patients affected by COVID-19. Ongoing clinical trials with SASP inhibitors targeting IL-6 as well as with ruxolitinib (NCT04331665) may provide useful information in this field. This research could eventually pave the way to exploration of whether other senotherapeutics, such as senolytics (compounds that selectively kill senescent cells) [90,91], or compounds able to promote clearance of senescent cells by the immune system [16,92], may eventually provide an additional advantage in the COVID-19 therapy.

Regarding this last aspect, T-cells engineered to express the NKG2D chimeric antigen receptor (CAR), which recognizes NKG2D ligands on the surface of SCs, may be used to target senescent cells [16,35]. In turn, the recent discovery of dipeptidyl peptidase 4 (DPP4) as a selective membrane marker of cellular senescence may provide a target for the development of immune-mediated clearance of senescent cells [16,93]. However, senescence immune surveillance in the lung may work through specific and different mechanisms than those reported for other tissues [37]. Hence, addressing this strategy in the case of infections with a lung target may be very challenging.

In the case of senolytics, it should be also addressed if the mechanism used to kill senescent cells can favor the release of intact viral particles in the environment. Preliminary results indicating the efficacy of the senolytic azithromycin in COVID-19 deserve particular attention. In the original paper describing the senolytic activity of this drug, an effect on mitochondria and mitochondrial oxygen consumption rate was reported [21], which are well known metabolic targets to induce apoptosis in senescent cells [94,95]. Azithromycin can also induce apoptosis by down-regulating Bcl-xL [96], which is a target of various senolytic compounds including ABT-263, ABT-737 and B1331852 [16]. So far, there has not been much attention paid to the mechanism of action of senolytics, as the goal of most research in this area is aimed at removing senescent cells independently from the mechanism of action. Necrosis, necroptosis, apoptosis and other subcategories of processes leading to cell death play an important and differential role in the host response to different viral infections [97]. This is an additional challenge, as it should be carefully evaluated if the mechanism inducing senescent cell death could eventually favor—or not—the spreading of the virus into neighboring cells. In this context, it might be useful to employ the recently proposed general term of ‘senocidals’ [90], to identify the pharmaceutical class of drugs that selectively kills senescent cells, and to discriminate between compounds that selectively eliminate senescent cells by apoptosis (‘senoptotics’) or by non-apoptotic means (‘senolytics’). In the case of elderly (comorbid) patients, a clean elimination of infected senescent cells may be critical for the termination of viral infection without side effects. While this area of research is completely unexplored, we deem that addressing these aspects could lead to the development of a wide range of therapeutic strategies to ameliorate the consequences associated with various viral infections.

Another aspect that deserves particular attention is that senotherapeutic molecules have off-target effects [98] and this is especially true for elderly or comorbid patients, where senotherapy side-effects due to systemic dosing could actually exacerbate the virally induced disease. Systemic delivery is currently the only means available for these compounds, for which we have convincing preclinical data from rodent studies [16]. However, there is a need to develop more-targeted delivery approaches so that senotherapeutic compounds can reach the senescent cells in the intended tissue site. The recent development of delivery systems based on encapsulation of diagnostic or therapeutic agents with β(1,4)-galacto-oligosaccharides and their preferential delivery to lysosomes of senescent cells is a step forward towards this intent [99]. Notably, this approach was effective in-vivo against pulmonary fibrosis.

**Figure 2:**
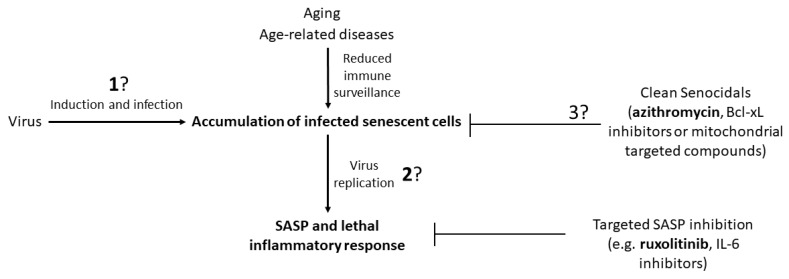
A schematic representation of the strategic therapies based on senotherapeutics that may be useful in the treatment of viral infections. Evidence from ongoing clinical trials point at azithromycin and ruxolitinib as major candidates in this field. Three aspects that need to be clarified by research before addressing the proper therapy are highlighted by question marks: (1) Information on the capacity of the virus to induce senescence and if the virus displays a preference to replicate in non-senescent versus senescent cells. (2) Understanding if viral replication is suppressed by cellular senescence or if the virus evolved mechanism(s) to bypass or use senescence to enhance replication. (3) In the case of strategies based on senocidals, it should be clarified if there are drugs or similar therapies that can induce cell death without favoring the spreading of the virus to neighboring cells. Selective Bcl-xL inhibitors and mitochondrial targeted compounds are likely candidates to induce a ‘clean’ apoptosis, avoiding the spreading of viral particles.

The potential therapeutic strategies and the key points that need to be addressed to pave the way to these strategies are shown in Figure 2. This field of research could be important, not only to cope with the current SARS-CoV-2 emergency, but also for future emergencies related to new or mutated viruses for which we are not fully prepared and for which there are no vaccines available.

## Figures and Tables

**Table 1:** Proposed therapies for COVID-19 targeting senescent cells or their signaling molecules.

Agent	Study Related to COVID-19 Therapy	Target/Pathways Related to Cellular Senescence	References
Tocilizumab (atlizumab)	Clinical trial (ChiCTR2000029765)	Blocker of IL-6R, IL-6 is among the most common SASP factor	[28,29]
Hydroxychloroquine and azithromycin	Clinical Trial (NCT04322396) and other pilot studies	Azithromycin exerts senolytic activity of human senescent lung fibroblasts; Hydroxychloroquine induces senescence in endothelial cells	[18,20,21]
Ruxolitinib	Bioinformatic study (supported by BenevolentAI) Clinical trial (NCT04331665)	JAK inhibitor, SASP suppressor	[15,16,17]
Rapamycin	Network-based drug repurposing methodology for HCoV	Inhibitor of mTOR, SASP suppressor	[12,13]
Melatonin	Network-based drug repurposing methodology for HCoV	SASP suppressor in lung fibroblasts	[12,14]

HCoV: Human coronaviruses; IL-6R: Interleukin-6 receptor; mTOR: mammalian target of rapamycin; SASP: senescence-associated secretory phenotype; JAK: Janus kinases.
